# The Maintenance of Mitochondrial DNA Integrity and Dynamics by Mitochondrial Membranes

**DOI:** 10.3390/life10090164

**Published:** 2020-08-26

**Authors:** James Chapman, Yi Shiau Ng, Thomas J. Nicholls

**Affiliations:** 1 Wellcome Centre for Mitochondrial Research, Faculty of Medical Sciences, Newcastle University, Newcastle upon Tyne NE2 4HH, UK; Yi.Ng@newcastle.ac.uk; 2 Biosciences Institute, Faculty of Medical Sciences, Newcastle University, Newcastle upon Tyne NE2 4HH, UK; 3 Translational and Clinical Research Institute, Faculty of Medical Sciences, Newcastle University, Newcastle upon Tyne NE2 4HH, UK

**Keywords:** mitochondria, mtDNA, cristae, mitochondrial fission, mitochondrial fusion, mitochondrial diseas

## Abstract

Mitochondria are complex organelles that harbour their own genome. Mitochondrial DNA (mtDNA) exists in the form of a circular double-stranded DNA molecule that must be replicated, segregated and distributed around the mitochondrial network. Human cells typically possess between a few hundred and several thousand copies of the mitochondrial genome, located within the mitochondrial matrix in close association with the cristae ultrastructure. The organisation of mtDNA around the mitochondrial network requires mitochondria to be dynamic and undergo both fission and fusion events in coordination with the modulation of cristae architecture. The dysregulation of these processes has profound effects upon mtDNA replication, manifesting as a loss of mtDNA integrity and copy number, and upon the subsequent distribution of mtDNA around the mitochondrial network. Mutations within genes involved in mitochondrial dynamics or cristae modulation cause a wide range of neurological disorders frequently associated with defects in mtDNA maintenance. This review aims to provide an understanding of the biological mechanisms that link mitochondrial dynamics and mtDNA integrity, as well as examine the interplay that occurs between mtDNA, mitochondrial dynamics and cristae structure.

## 1. Introduction

Mitochondria act as metabolic hubs within the cell to facilitate a myriad of essential cellular processes such as energy production, the regulation of apoptosis and cellular signalling pathways, amongst others. They are unique organelles in the fact that they harbour their own genome that is distinct from the nuclear genome. In human cells, this consists of a circular double-stranded DNA molecule, referred to as mitochondrial DNA (mtDNA). A typical cell possesses between a few hundred and several thousand copies of mtDNA that are replicated independently of the cell cycle within the mitochondrial matrix and segregated between mitochondria. These genomes are closely interlinked with the cristae ultrastructure of the mitochondrion. Once they have been replicated, mtDNA molecules are subsequently distributed around the mitochondrial network by processes that rely on the plastic nature of mitochondria to undertake fission and fusion events, as well as the modulation of cristae structure. Defects in the fission and fusion machinery, or in proteins associated with modulating cristae structure, disrupt the even allocation of mtDNA throughout the network and subsequently to daughter organelles and cells. Defects in mtDNA metabolism typically manifest as the accumulation of mtDNA molecules harbouring deletions and/or as a depletion in the number of copies of mtDNA per cell. The close association between mtDNA and cellular energy production means that the loss of mtDNA number and integrity limits the capacity for the mitochondria to meet the energy demands of an organism. From a clinical perspective, patients carrying mutations within dynamics or cristae-associated genes display heterogeneous neurological phenotypes. There is a clear requirement to understand the basic biological processes linking mitochondrial structure with mtDNA maintenance and its links to mitochondrial disease.

This review aims to disentangle the relationships that exist between mitochondrial dynamics, cristae structure and the organisation of mtDNA. In addition, the biological mechanisms that may prompt the disruption of mtDNA integrity following the impairment of mitochondrial dynamics are assessed. Finally, these mechanisms are discussed in context with observations from the clinic.

## 2. Mitochondrial DNA

### 2.1. The Origins of mtDNA

mtDNA is present in the mitochondria of almost all eukaryotic organisms, and the advent of genome sequencing has allowed its evolutionary origins to be dissected. It is now understood that mtDNA derives from an event whereby a host cell engulfed an alphaproteobacterium [1]. This endosymbiotic occurrence fostered a relationship in which the bacterium was utilised for its energy-producing capacity by the host cell. This gave rise to the first complex eukaryotic cells, and since then the course of evolution and the transfer of mitochondrial genes to the nucleus has led to mitochondrial genomes that vary significantly in both structure and size between modern eukaryotic organisms. For example, higher plants harbour genomes that are typically 200–300 kb in size made up of linear and small circular regions of DNA, whereas, algae and fungi have much smaller linear genomes in the region of 30–90 kb [2,3].

**Figure 1:**
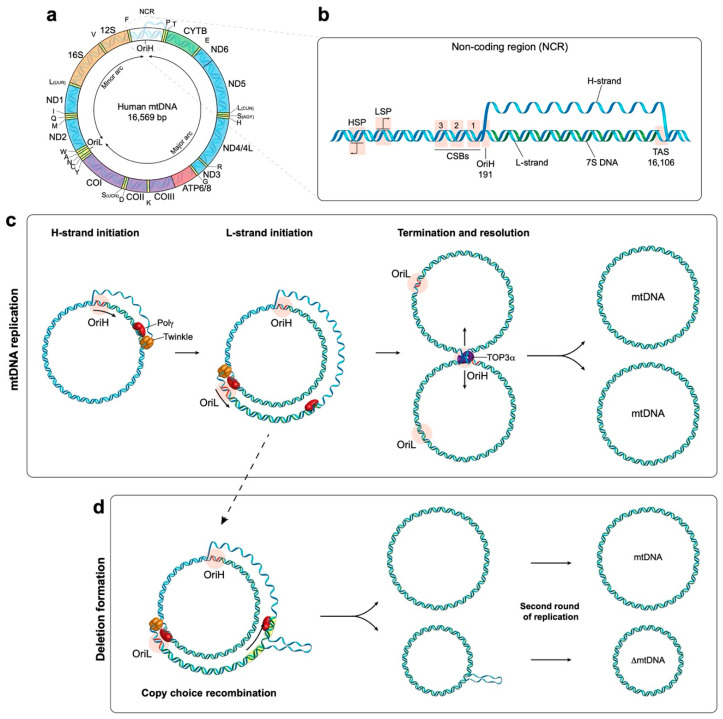
Schematic overview of the human mtDNA genome, its replication and the formation of deletions. (**a**) The structure of mtDNA highlighting the arrangement of protein coding genes, rRNAs (orange) and tRNAs (yellow). The replication origins of the heavy and light strand (OriH and OriL, respectively) are highlighted. (**b**) Enlargement of the mtDNA non-coding region (NCR) depicting the arrangement of the heavy strand promoter (HSP) and light strand promoter (LSP), the three conserved sequence boxes (CSB), OriH and the termination-associated sequence (TAS). The premature termination of the DNA synthesis of the H-strand at TAS results in the formation of a triple-stranded displacement-loop structure termed the D-loop. The short double stranded product formed within the D-loop is termed 7S DNA. (**c**) mtDNA replication is initiated at OriH and proceeds unidirectionally until OriL is reached. At this point, DNA synthesis of the light strand is initiated, and both strands are synthesised simultaneously until two completely replicated genomes are produced. The two replicated genomes are physically interlinked by a single-stranded overlap structure, termed a hemicatenane. This structure is resolved by topoisomerase 3α (TOP3α) to produce two separate mtDNA molecules. (**d**) Copy choice recombination model for the formation of mtDNA deletions. mtDNA deletions generally occur in the major arc. The replication of a repeat sequence in the template heavy strand (yellow boxes) can lead to stalling of POLγ which results in its dissociation from the newly synthesised DNA-end. When Polγ reanneals, it may associate at another repeat sequence further along the template. Following the completion of replication, this slippage event produces two mtDNA genomes; one full length molecule and a second heteroduplex molecule (which has a full-length heavy strand alongside a deletion-containing light strand). The subsequent replication of the heteroduplex molecule culminates in the formation of mtDNA harbouring the deletion.

Human mtDNA is 16,569 base pairs in length and is organised into a double-stranded circular structure containing 37 genes which encodes for 13 mitochondrial proteins (Figure 1a) [4]. These proteins are all essential components of the oxidative phosphorylation (OXPHOS) system. The OXPHOS machinery is made up of four respiratory chain complexes and the ATP (adenosine triphosphate) synthase which are responsible for energy production in the currency of ATP [5]. In addition, mtDNA encodes 22 transfer RNA (tRNA) and 2 ribosomal RNA (rRNA) molecules which are components of the mitochondrial translation system [4]. The rest of the mitochondrial proteome, currently estimated at 1158 proteins [6], is encoded by the nucleus as the result of the lateral transfer of mitochondrial genes [1]. This evolutionary pressure towards mtDNA reduction means that human mtDNA possesses very few noncoding regions and contains areas of overlapping genes [4]. The OXPHOS system is made up of approximately 90 proteins, and as such is comprised of the products of both mitochondrial and nuclear genes [7]. This dual-genetic origin requires that nuclear-encoded subunits be translated in the cytosol prior to being imported into the mitochondria. Conversely, subunits encoded by mtDNA are synthesised and assembled within the mitochondria by a dedicated mitochondrial translation machinery. Once the nuclear subunits are imported, they are assembled alongside the mtDNA-encoded subunits to form the respiratory complexes that make up the electron transport chain.

### 2.2. mtDNA Replication and Segregation

Unlike nuclear DNA, the replication of mtDNA occurs throughout the cell cycle. The process of mtDNA replication utilises a different set of proteins to those which carry out replication of the nuclear genome [8]. These proteins are all nuclear-encoded and are imported into the mitochondrial matrix from the cytosol. The mitochondrial transcription machinery generates the primers for mtDNA synthesis and can therefore be considered essential components of the mtDNA replication machinery. The mechanisms of replication will be outlined in brief here; for further details there are a number of excellent reviews on the topic [9,10].

#### 2.2.1. mtDNA Transcription

Human mtDNA consists of a ‘heavy’ (guanine rich) and a ‘light’ (cytosine rich) strand, with each strand having a single promoter region located close together within the noncoding region (NCR) of the genome (Figure 1b) [11]. These are referred to as the light strand promoter (LSP) and heavy strand promoter (HSP) respectively and facilitate polycistronic transcription of both strands of mtDNA almost in their entirety. Transcription initiation minimally requires the mitochondrial RNA polymerase (POLRMT) with mitochondrial transcription factor A (TFAM) and mitochondrial transcription factor B2 (TFB2M) [12,13]. Following initiation, the mitochondrial transcription elongation factor TEFM promotes POLRMT processivity [14,15]. RNA primers responsible for the initiation of DNA synthesis are generated when transcription from the LSP is terminated prematurely around a series of conserved sequence blocks (CSBs) downstream of LSP, forming an R-loop [16]. This allows the DNA synthesis machinery to assemble and leads to the initiation of DNA synthesis from OriH [17,18].

#### 2.2.2. mtDNA Replication

The protein complex responsible for DNA synthesis is termed the replisome. At the core of the replisome is DNA polymerase-γ (POLγ) which is responsible for synthesising the DNA [19]. POLγ is a heterotrimer made up of a catalytic subunit (POLγA) which functions as a highly accurate proofreader of the newly synthesised DNA [20]. The processivity of POLγA is increased by two copies of the accessory subunit (POLγB) which interact with the DNA substrate [21,22]. In order for POLγ to access and replicate the DNA, it is necessary for the dsDNA to first be unwound. This is performed by the DNA helicase TWINKLE which travels in front of the replication fork and unwinds DNA in the 5′ to 3′ direction [23]. The activity of both TWINKLE and POLγ is enhanced by the mitochondrial single stranded DNA-binding protein (mtSSB) [24]. mtSSB has also been demonstrated to bind to and stabilise the unwound single stranded DNA behind the replication fork [23,24].

During the initial phase of DNA synthesis only the H-strand is replicated. Once the replisome has travelled approximately 12,000 bp it reaches the origin of L-strand replication (OriL), which becomes single stranded and folds into a stem-loop structure [25]. This structure prevents mtSSB from binding and provides a stretch of poly(T) ssDNA that is accessible to POLRMT [26,27]. POLRMT transcribes a short 25–75 bp primer on the single-stranded template, from which synthesis of the L-strand is initiated [27]. See Figure 1c for a schematic overview of mtDNA replication. The strand displacement model proposes that the long tract of exposed H-strand ssDNA is coated and protected by mtSSB until DNA synthesis is initiated from OriL. This model has been reviewed in depth elsewhere [9]. In addition to this model, the bootlace model proposes that the displaced single-stranded lagging-strand template DNA is instead coated by RNA transcripts [28]. Fully double-stranded replication intermediates reminiscent of coupled leading and lagging-strand DNA replication have also been observed and characterised using two-dimensional agarose gel electrophoresis [29,30,31].

#### 2.2.3. Termination of mtDNA Replication

Once DNA synthesis is complete, the primers at OriL and OriH must be removed to allow for ligation of the DNA ends. Studies have revealed that RNase H1 is involved in the process of primer removal as the loss of RNase H1 in mouse embryonic fibroblasts (MEF) leads to the retention of the primers at sites including both origins of replication; OriL and OriH [32]. The role of RNase H1 in removing primers has been reconstituted in vitro at OriL [33]. Furthermore, the loss of RNase H1 in vivo in mice is embryonically lethal as a consequence of significant mtDNA loss [34]. Following primer removal, the DNA ends need to be prepared to allow ligation by DNA ligase III to occur [35,36]. In a reconstituted system, the mitochondrial genome maintenance exonuclease 1 (MGME1) protein facilitates efficient ligation by modifying single stranded DNA overhangs that occur on the 5′ end of the newly synthesised DNA following primer removal [36]. The loss of MGME1 in vivo has been shown to result in diminished ligation at OriH [37].

Once replication is complete, the two genomes remain connected at the OriH region by a single stranded linkage (termed a hemicatenane) and must be separated (see Figure 1c for a schematic overview). Recent work has revealed that topoisomerase 3α, a type 1A topoisomerase, cleaves the single strand linkage to allow passage of one of the strands to occur, resulting in the separation of the two mtDNA molecules [38].

#### 2.2.4. Formation of mtDNA Deletions

Common manifestations of defects in mtDNA maintenance are deletions and rearrangements of mtDNA. Single, large-scale deletions can cause mitochondrial DNA diseases if they undergo clonal expansion to accumulate beyond a biochemical threshold, typically 60–90% [39]. Such deletions are sporadic and believed to be formed during embryonic development. The most well-studied single deletion is the 4977 bp “common deletion”, underlying Pearson’s syndrome and Kearns–Sayre syndrome in early life, and chronic progressive external ophthalmoplegia (CPEO) in later life [40]. Alternatively, multiple mtDNA deletions can occur secondarily to disease-causing mutations in nuclear genes that encode factors involved in mtDNA replication, nucleotide metabolism and mitochondrial dynamics [41]. Multiple deletions are also observed in post-mitotic tissues during normal ageing [42,43]. Both the mechanism of deletion formation and the mechanism of clonal expansion of deletions have been the subject of debate. The clonal expansion of mtDNA deletions has been recently reviewed extensively elsewhere [44].

The formation of mtDNA deletions has been proposed to occur either during mtDNA replication or as the result of double-strand breaks [45]. An early model proposed a slip-replication mechanism for the formation of the common deletion [46]. This model involves the annealing of the displaced H-strand to a downstream repeat sequence in the leading-strand template, leading to the removal of the sequence between the two repeats [46]. A more recent model that is supported by in vitro reconstitution experiments suggests that deletions are the result of copy-choice recombination [45,47]. Deletions in mtDNA predominantly form in the major arc between OriH and OriL in the direction of replication. During the synthesis of the L-strand, replication slippage can occur. Specifically, the 3′ end of the nascent L-strand becomes dissociated from the H-strand template at one repeat sequence, and subsequently reanneals to another repeat sequence further along the template. This produces a heteroduplex molecule consisting of a complete H-strand and a shortened L-strand harbouring the deletion. Subsequent rounds of replication will produce shortened mtDNA molecules containing the deletion, see Figure 1d [47].

It has also been observed that inducing high levels of double-strand breaks in mtDNA, which are normally rapidly degraded [48], can result in the formation of deletions [49,50]. Therefore, it has been suggested that limited nucleolytic processing of double-strand breaks could lead to the annealing of repeat sequences and the generation of deletion-containing mtDNA molecules [45,51]. As mitochondria have not been found to possess repair pathways for double-strand breaks comparable to those in the nucleus, this mechanism would presumably operate by a distinct mechanism.

### 2.3. mtDNA Packaging

A typical mammalian cell harbours between 1000 and 10,000 copies of the mtDNA genome that exist in a DNA-protein complex, termed a nucleoid [52]. Studies using super-resolution microscopy have revealed that nucleoids generally contain a single copy of mtDNA and have a diameter of approximately 100 nm [53,54]. This small size is attributed to the compaction of mtDNA by TFAM. Specifically, TFAM binds along the length of mtDNA at a ratio of 1 subunit per 16–17 bp, instigating bending and loop formation along the DNA backbone that results in compaction [55,56,57,58]. The stable protein filaments that are formed prevent POLRMT and TWINKLE from accessing the DNA [52,55]. Reconstituted nucleoids in vitro were demonstrated to become progressively more compact with increasing levels of TFAM, and a wide range of packaging densities were observed at typical physiological concentrations of TFAM [52]. It has therefore been speculated that TFAM may regulate the overall transcription and replication rate of mtDNA by controlling its accessibility to relevant proteins. TFAM is the most abundant protein found associated with nucleoids, however, a number of proteins related to replication, transcription and translation are also commonly found in association with nucleoids [59,60,61]. It has been proposed that these proteins are located within the “inner core” of the nucleoid, whereas other proteins such as mitochondrial chaperones and membrane binding proteins constitute the peripheral layer [62].

## 3. Membrane Dynamics and the Organisation of mtDNA

Once the mitochondrial genomes have been separated, they need to be segregated and dispersed around the mitochondrial network. Because the OXPHOS complexes are composed of both mtDNA-encoded and nuclear-encoded subunits, the complexes are assembled in situ proximal to the nucleoid [63,64]. The capacity for mtDNA to spatially diffuse by itself is limited and so nucleoids require mitochondrial membrane structure and dynamics to aid in their distribution around the mitochondrial network [65]. The disruption of nucleoid distribution can lead to a mosaic pattern of respiratory activity, in which only regions of the mitochondrial network that contain nucleoids are capable of assembling OXPHOS complexes and are therefore capable of oxidative ATP production [66]. Structurally, mitochondria are composed of two phospholipid membranes arranged as an outer mitochondrial membrane (OMM) and inner mitochondrial membrane (IMM). The space between these two membranes is referred to as the intermembrane space (IMS). The IMM is intricately folded to form the cristae structures that harbour the respiratory chain complexes. The interior space enclosed by the IMM is referred to as the matrix and contains the mitochondrial genome. mtDNA is closely associated with the IMM and is suggested to be physically attached, a relationship that likely aids in its distribution [67,68,69]. Early studies identified a protein complex bound to the OriH region of mtDNA and to the IMM, although the factors responsible were not identified [67,70]. It has since been found visually using electron microscopy and immuno-gold labelling that mtDNA is found in close apposition to the IMM [69]. A number of plausible candidates for mtDNA tethering are discussed later in this review. This section aims to evaluate how the dynamic nature of mitochondrial membranes facilitate the distribution of the replicated genomes around the network.

### 3.1. Mitochondrial Fission and Its Role in mtDNA Distribution

Mitochondria cannot be synthesised de novo; they must have the capacity to grow and divide in order to distribute the replicated genomes to daughter mitochondria. The appearance of mitochondria within a cell can vary significantly; they can exist as isolated entities or be fused together in vast sprawling networks [71]. This versatile nature allows them to sustain energy production as well as act as signaling platforms in complex cellular processes such as apoptosis, autophagy and senescence [72,73,74]. There are two main mechanisms that underlie mitochondrial dynamics: fission and fusion. First, the relationship between fission and mtDNA distribution and maintenance will be discussed.

#### 3.1.1. Mitochondrial Fission

Mitochondrial fission is the division of a mitochondrion into multiple distinct mitochondria and has been implicated in the distribution of nucleoids around the mitochondrial network [75,76,77]. Fission requires the progressive constriction, and eventual scission, of the IMM and OMM. The initial constriction occurs at contact sites between the OMM and the endoplasmic reticulum (ER), where actin polymerisation provides the force required to contract the mitochondrial membrane [78,79,80]. Further constriction is primarily fulfilled by dynamin-related protein 1 (DRP1), a cytosolic protein that translocates to the OMM and interacts with several adaptor proteins [81]. DRP1 binds with mitochondrial fission factor 1 (MFF), mitochondrial dynamics protein of 49kDa (MID49), MID51 and mitochondrial fission 1 protein (FIS1) [82,83,84,85]. Utilising GTP (guanine triphosphate), polymerisation of DRP1 with MID49 and MID51 occurs leading to the formation of linear filaments. GTP hydrolysis induces the oligomerisation of DRP1 and filament shortening to create rings which constrict around the mitochondria [79,86]. It has now been established that DRP1-mediated constriction is sufficient for the final scission step to separate mitochondria [87,88]. Recent studies have also highlighted the importance of additional interorganelle contacts for mitochondrial fission, with roles for lysosomes in fission regulation and with Golgi-derived vesicles during final scission [89,90].

It has been observed that ER-OMM contact sites that mark sites of mitochondrial division are often also spatially located adjacent to replicated nucleoids [75,76,77], suggesting a role for fission in mtDNA segregation. Furthermore, the visualisation of DRP1 and MFF using confocal microscopy has demonstrated colocalisation at sites adjacent to the nucleoid [69,77,91]. Where division occurs between replicated nucleoids, the daughter mtDNA molecules are subsequently observed to be located at the tips of the separated mitochondria [75,76]. This mechanism ensures that following division each mitochondrion receives a copy of the genome, and secondly functions to disperse nucleoids throughout the mitochondrial network. At this stage, it is unclear what signalling takes place to ensure that division occurs between the two replicated nucleoids.

#### 3.1.2. Defects in Mitochondrial Fission and Human Disease

At this point in time, the prevalence of human diseases secondary to defects in mitochondrial fission is not known. However, they would appear to be much less common than diseases that are related to mitochondrial fusion. The most common clinical manifestations that occur in relation to disruption of mitochondrial fission genes are subtypes of Charcot–Marie–Tooth (CMT) neuropathy and optic neuropathy, as identified following mutations that occur in *DRP1*, *GDAP1*, *INF2*, *MFF* and *SLC25A46* [92,93,94,95,96]. Severe neurological presentations, such as neurodevelopmental delay and epilepsy, are observed following mutations of the *DRP1* gene [97]. Leigh-like syndrome has been observed in patients harbouring mutations in *MFF* and *SLC25A46* [98,99]. Mutations in *DNM2* have been linked to CPEO and central core myopathy [100,101], a form of myotubular myopathy. Extra-neurological involvements are relatively uncommon, except cardiac arrhythmia and neutropenia, which have been associated with mutations in *DNM2* [101,102], and glomerular disease, described in INF2-related disease (Appendix A Table A1).

Mitochondrial respiratory chain deficiencies and multiple mtDNA deletions have been demonstrated in muscle biopsies taken from patients harbouring *DNM2* mutations [103]. Normal qualitative and quantitative assessments of mtDNA were reported in the skeletal muscle or fibroblasts that contain mutant forms of other mitochondrial fission-associated genes such as *GDAP1*, *INF2* and *MFF* (Table A1) [93,94,98,104,105,106,107,108,109]. These observations would suggest that the biological consequences of these genetic defects are tissue specific, given that only *DNM2* mutations primarily manifest with a myopathic phenotype [100,101]. Whilst other fission-associated genes predominantly cause peripheral neuropathy including optic neuropathy or CNS involvement, it is important to note that these affected tissues are far less accessible for further characterisation compared to muscle biopsy.

#### 3.1.3. Mitochondrial Fission and mtDNA Integrity

The disruption of mitochondrial fission by genetic knockdown of the fission factors *DRP1* or *MFF* results in an elongated and fused mitochondrial network that prevents the even distribution of nucleoids around the network, resulting in the clustering of nucleoids [66,77]. Reports in the literature differ on its effects on mtDNA copy number and respiratory activity. In human cells, one study reported that the loss of *DRP1* induced mtDNA loss and a reduction in respiratory activity as measured by ATP production [110], while a separate study found that the knockdown of *DRP1* or *MFF* using siRNA did not affect the overall mtDNA copy number [77]. In mice, mtDNA copy number and respiratory function were shown to be unaffected in *Drp1* knockout MEFs [111], but a whole-body knockout of *Drp1* is embryonically lethal [111,112]. In a tissue-specific knockout of *Drp1* in the heart, mice survived for 11 days and displayed exacerbated mitochondrial fusion, nucleoid clustering, reduced mtDNA and respiratory defects [66]. Interestingly, in this model immunofluorescent staining of the mtDNA-encoded cytochrome c oxidase 1 subunit (COX1) revealed that there was an increased staining intensity in regions where nucleoids were clustered together, and a decreased intensity in areas with a sparse presence of nucleoids [66]. These data suggest that nucleoid clustering leads to a mosaic pattern of respiratory subunit distribution in the mitochondrial network. Similarly, the knockout of *MFF* is associated with premature death and defective mitochondrial respiratory activity [113]. In some cases, it was reported that *DRP1* knockout was associated with enlarged nucleoid size [77]. However, this may be a limitation associated with the microscope resolution used, as it would not be expected that fission would affect the decatenation of the DNA molecules. As such, these apparently enlarged nucleoids may represent decatenated mtDNA molecules located in close proximity, which are beyond the limits of detection, although this remains to be confirmed.

**Figure 2:**
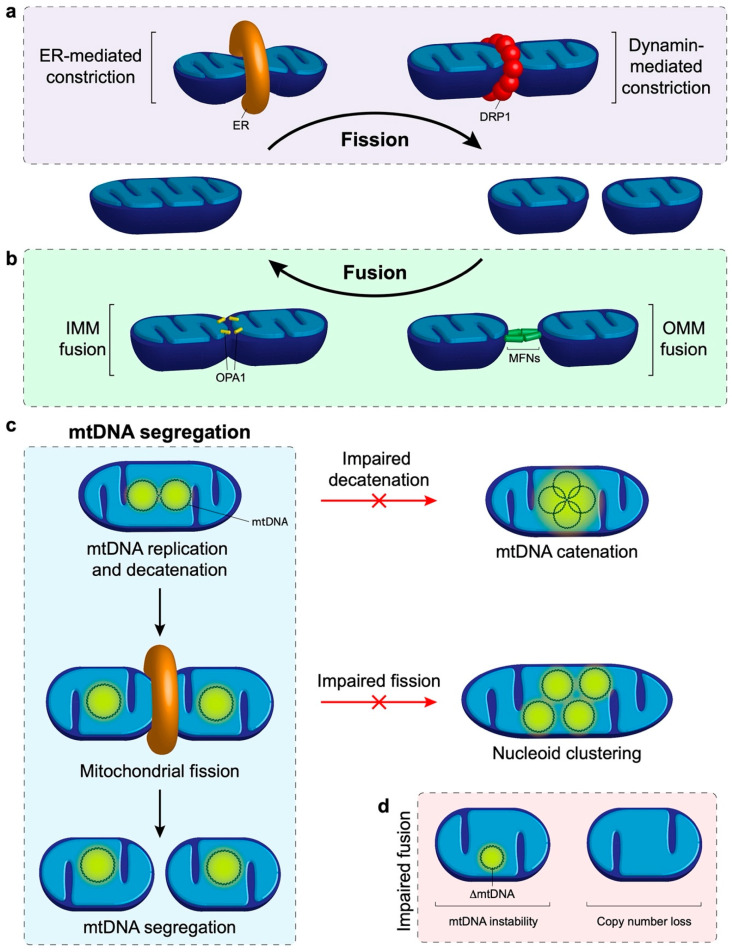
Mitochondrial dynamics and its role in mtDNA distribution. (**a**) Schematic overview of mitochondrial fission. Constriction of the mitochondrion occurs by the endoplasmic reticulum (ER) and dynamin related protein 1 (DRP1). The final scission step is performed by DRP1 to produce multiple independent mitochondria. (**b**) Mitochondrial fusion outline. The mitofusins (MFNs) are responsible for tethering neighbouring mitochondria and fusing the outer membranes together. Fusion of the inner mitochondrial membrane is mediated by optic atrophy 1 (OPA1). (**c**) Summary of mtDNA segregation and distribution. First, newly replicated mtDNA molecules that are joined by a hemicatenane must be physically separated by topoisomerase 3α. Failure of this process results in the formation of multiple physically joined mtDNA genomes, termed mtDNA catenanes. Following separation, mitochondrial fission is required to distribute the replicated nucleoids into separate mitochondria and facilitate distribution of the genomes around the mitochondrial network. An impairment of fission results in a clustered phenotype whereby many replicated nucleoids are observed in close proximity to each other but are not physically linked together. (**d**) A lack of mitochondrial fusion is associated with mtDNA instability as observed by the progressive onset of mtDNA deletions and point mutations. In addition, the lack of fusion leads to a depletion in mtDNA copy number.

Cardiolipin is an integral structural component of mitochondrial membranes that is synthesised from phosphatidic acid (PA). The IMM contains approximately 20% cardiolipin, the presence of which is considered a signature of the IMM. Both cardiolipin and PA have been implicated in facilitating the fission and fusion processes (this topic has been reviewed in depth elsewhere [114]). Briefly, DRP1 binds to both cardiolipin and PA, cardiolipin at the OMM can stimulate oligomerisation of DRP1 and subsequent GTP hydrolysis to induce fission [115,116,117]. It has also been shown that DRP1 binding to cardiolipin induces reorganisation of the membrane to an inverted hexagonal, nonbilayer configuration that promotes membrane constriction [118]. Conversely, the activity of DRP1 can be restrained at the OMM by the reversion of cardiolipin to PA by MitoPLD (mitochondria-localised phospholipase D), as the enhanced level of PA inhibits oligomerisation-stimulated GTP hydrolysis that is responsible for membrane constriction [119]. As such, the loss of cardiolipin is associated with a reduced capacity to correctly segregate and guide nucleoids to the daughter mitochondria; this leads to a lack of mtDNA inheritance between replicating cells, resulting in a dysfunctional respiratory phenotype in daughter cells [120,121,122]. Modulating the level of cardiolipin has also been associated with loss of mtDNA and subsequent mitochondrial dysfunction [121,123]. Clearly, fission plays an important role in facilitating the dissemination of mtDNA around the mitochondrial network and to subsequent daughter organelles (see Figure 2a for a schematic overview). Furthermore, these observations also highlight how mitochondrial fission maintains respiratory function independently of mtDNA copy number or integrity. It is clear that if the fission process is not tightly regulated then cellular respiration will be affected and can contribute to the mitochondrial disease phenotypes discussed earlier.

### 3.2. Mitochondrial Fusion and Its Role in mtDNA Maintenance and Distribution

Together with mitochondrial fission, mitochondrial fusion has been implicated in the dissemination of nucleoids around the network. Furthermore, fusion is recognized as an important mediator of mtDNA maintenance. Defects in mtDNA maintenance may manifest as either a quantitative reduction in mtDNA copy number (depletion) or as an accumulation of rearranged mtDNA molecules (deletions and/or duplications), all of which may be indicative of impaired mtDNA replication. Defects in the proteins that regulate mitochondrial fusion dynamics have been implicated in a range of genetic diseases that will be discussed here and are summarised in Table A1. Their contribution to the development of disease will be described in relation to their role in mtDNA maintenance.

#### 3.2.1. Mitochondrial Fusion

Mitochondrial fusion is the joining of two separate mitochondria and is important to enable the sharing of contents between neighbouring mitochondria [124]. The fusion of the OMM and IMM occur sequentially, therefore the outer membranes are joined first. This is regulated by two outer membrane proteins, the dynamin related GTPases mitofusin 1 & 2 (MFN1 and MFN2). MFN1 is responsible for tethering the adjoining mitochondria together in a GTP-dependent dimerization process, prompting a conformational change which in turn mediates GTP hydrolysis by MFN1 to pull the membranes together, resulting in mitochondrial fusion [125,126,127].The role of MFN2 is less clear, although it has been implicated in interactions between mitochondria, as well as between mitochondria and the ER [128,129,130]. Following OMM fusion the IMM can be fused. IMM fusion is controlled by optic atrophy 1 (OPA1). OPA1 exists in multiple forms of different sizes which are regulated by proteolytic processing; long OPA1 is IMM-anchored while the short OPA1 is soluble. The presence of OPA1 in its long isoform has been associated with promoting IMM fusion [131]. Processing of OPA1 to its short isoform either by YME1L1 or OMA1 is associated with mitochondrial fission [132,133].

There is evidence that OMM fusion proteins also play a role in the dissemination of nucleoids. It has been reported that the knockout of OMM fusion proteins *Mfn1 & 2* in MEFs leads to the clustering of nucleoids [134]. Using super-resolution microscopy these clusters were confirmed to be separate individual nucleoids in close proximity rather than a cluster of interlinked DNA molecules. The role of IMM fusion in nucleoid distribution is less clear. It has been reported by some groups that the loss of *OPA1* is associated with a reduction in the number of nucleoids per cell, and in some cases a clustered phenotype [77,135,136]. In contrast, a recent study found that in MEFs the removal of *Opa1* was not associated with any alterations in mtDNA distribution, although there was a reduction in copy number [134]. This normal nucleoid distribution following *Opa1* knockout may be explained by the role of MFN2 in forming a tether between the OMM and ER [137,138]. As discussed earlier, it is recognized that the ER localises at positions of nucleoid replication and is implicated in coordinating mitochondrial constriction at these sites [75]. Taken together, this highlights the importance of the OMM acting as a signalling platform for coordinating the circulation of nucleoids around the mitochondrial network.

Interestingly, the knockdown of *MFN1 & 2* in conjunction with *DRP1* has been found to prevent the clustering of nucleoids in human cells [77]. Mechanistically, how the impairment of both fission and fusion would restore nucleoid distribution is not clear as it may be expected that this would render the mitochondria unable to be dynamic, and thus unable to evenly distribute nucleoids. Another study in vivo demonstrated that the dual knockout of *Mff* and *Mfn1* in mice could completely rescue the heart dysfunction, shortened life span, and respiratory chain dysfunction associated with *Mff* knockout [113], although this rescue was tissue specific. It was suggested that this dual knockout reinstates a balance of fission and fusion, rather than hyperfusion or fragmentation. The relationship between mitochondrial dynamics, nucleoid segregation and maintenance of the respiratory chain is complex and therefore further study is still required to disentangle its intricacies. See Figure 2 for an overview of the interplay between fission and fusion in mtDNA distribution.

#### 3.2.2. Defects in Mitochondrial Fusion and Human Disease

In recent years, genetic defects involved in mitochondrial fusion have emerged to be the common cause of several neurological and ophthalmological disorders. *OPA1* mutations account for 60% of dominant optic atrophy (DOA) cases [139], and the prevalence of the disease has recently been revised to 1 in 34,000 [140]. More complex neurological phenotypes such as cerebellar ataxia, spasticity, CPEO [141,142] and more recently, Parkinsonism and dementia, have been identified in patients with *OPA1* mutations [143]. *MFN2* mutations are the fourth most common cause of CMT neuropathy [144,145], accounting for 20% of CMT2 [146], a form of dominant axonal neuropathy. Mutations in *SPG7* were initially described in hereditary spastic paraplegia associated with mitochondrial OXPHOS defects [147]. Independent cohort studies have subsequently shown that cerebellar ataxia could be the most prominent clinical feature without evidence of upper motor signs in cases of *SPG7* mutations, and are the most common or second most frequent cause of recessive genetic ataxia in European populations [148,149]. Interestingly, a recent Spanish study suggested that around one-fifth of SPG7 cases exhibited Parkinsonism [150].

AFG3L2 and SPG7 together form the subunits of the m-AAA metalloprotease complex, which is crucial for the maturation, maintenance and quality control of the mitochondrial proteome [151]. Heterozygous mutations in *AFG3L2* cause spinocerebellar ataxia type 28 (SCA28) and mitochondrial respiratory chain deficiency [151]. Given the close interaction between AFG3L2 and paraplegin (SPG7), there is little surprise that genetic defects result in many overlapping clinical features of neurodegeneration and the classic phenotypes of mitochondrial dysfunction such as CPEO and multiple mtDNA deletions in the muscle [152,153].

Severe, childhood-onset encephalopathy has been observed in mutations in *OPA1* (recessive), *FBXL4* and *YME1L1* (Table A1). On the other hand, mitochondrial DNA depletion is rare in defects of mitochondrial fusion and has only been identified in several cases of severe childhood disease secondary to mutations in *MFN2* and *FBXL4* [154,155]. Intriguingly, multiple lipomatosis, as previously observed in myoclonic epilepsy and ragged-red fibres (MERRF) syndrome but no other forms of primary mtDNA mutations, has been identified in several families of *MFN2* mutations [156,157,158].

#### 3.2.3. Mitochondrial Fusion and mtDNA Copy Number

A number of studies have highlighted that mitochondrial fusion is critical for the maintenance of mtDNA copy number. In yeast, the loss of fusion activity leads to a loss of mtDNA copy number [159,160]. Consistent with this observation, mtDNA content and respiratory activity is reduced in MEFs following the knockout of OMM fusion factors *Mfn1 &2* either alone or together or following knockout of the IMM fusion factor *Opa1* [134,135]. Mice carrying a mutation in *Opa1* display mtDNA loss and reduced mitochondrial function in the heart [161], while whole-body knockouts of MFN1 & 2 are embryonically lethal [162]. The roles of both MFN1 & 2 have therefore been studied in a tissue-specific context. Work in mice has shown that the knockout of *Mfn1 & 2* in heart and skeletal muscle leads to a drop in overall mtDNA copy number and OXPHOS deficiency [134,136]. Cardiolipin also has a role in regulating fusion as it is necessary for the biogenesis of OPA1 and for the formation of higher order OPA1 oligomers which are required for fusion [163,164,165]. The cardiolipin precursor PA has also been associated with the induction of fusion in an MFN dependent manner [166]. The disruption of cardiolipin levels is linked with a reduction in mtDNA copy number [121,123]. ATAD3 (ATPase family AAA-domain containing protein 3 A) has also been implicated in maintaining mitochondrial fusion as its manipulation either by knockdown or overexpression leads to mitochondrial fragmentation [167,168,169,170]. This fragmented phenotype is mediated by the recruitment of DRP1 to the OMM via oligomerisation of the ATAD3 coiled-coil domain [170]. Furthermore, it was demonstrated that in addition to activating DRP1, the dimerisation of ATAD3 provokes mtDNA instability by disrupting the binding of TFAM and mtDNA. Indeed, the knockdown of ATAD3 has been associated with a reduction in mtDNA content [170]. In human fibroblasts that are either deficient for ATAD3 or harbour a duplication of the ATAD3 gene cluster, nucleoids were found to be enlarged and clustered together suggesting a role of ATAD3 and fusion in mtDNA distribution [171,172]. The knockout of *ATAD3* in skeletal muscle has been associated with the progressive formation of mtDNA deletions and copy number depletion [173].

The mechanisms underlying the loss of mtDNA when fusion is impaired have long been unclear, although recent work has shed light on potential links between the two. In general, the loss of copy number indicates that there is a defect with the process of mtDNA replication. It has been demonstrated that mitochondrial fusion is necessary to facilitate high levels of replication through content mixing to ensure a proper stoichiometry of replisome components. The loss of OPA1 alone or the collective loss of MFN1 & 2 together prompts an imbalance of replisome factors and thus leads to a reduced rate of mtDNA replication, leading to mtDNA depletion [134].

#### 3.2.4. Mitochondrial Fusion and mtDNA Integrity

It has been observed that aside from being required to maintain copy number, fusion appears to play an important role in maintaining the integrity of the mitochondrial genome. For example, mutations in the *OPA1* gene have been associated with the accumulation of mtDNA deletions and OXPHOS defects in the skeletal muscle of patients [141,174,175]. Similarly, patients with mutations in the OMM fusion protein MFN2 display evidence of deletion-containing mtDNA genomes [154,176]. In animal models, the knockout of *Mfn1 & 2* in the skeletal muscle of mice is associated with an increased occurrence of point mutations and deletions of mtDNA [136]. On the other hand, another group studying the cardiac tissue of mice with MFN1 & 2 genetically removed found no differences in levels of mtDNA mutations or deletions [134]. These studies highlight that effects may be tissue-specific, and that care must be taken when comparing effects in patients carrying missense mutations with effects seen in knockout animal models. However, from a clinical perspective, mutations in the fusion machinery have a clear association with the progressive onset of mtDNA mutations and deletions (see Table A1).

Multiple lines of evidence indicate that fusion plays a protective role against mutations. Work using fibroblasts derived from *MFN2* patients found that these cells display a reduced capacity to repair mtDNA damage [176]. This finding potentially suggests that fusion may preserve mtDNA integrity by enabling the repair of DNA damage or by facilitating the clearance of mitochondria harbouring damaged DNA. In support of this notion, excessive mitochondrial fragmentation (which would occur with impaired fusion) has been associated with an increased production of reactive oxygen species [177]. Indeed, in an *Opa1* mutant mouse it was observed that there was an increased level of ROS coupled with a reduced antioxidant capacity [161]. A separate study used the mutator mouse, which has a mutation in the proofreading domain of polymerase *PolgA*, and consequently rapidly accumulates mtDNA mutations, crossed with a knockout of MFN1 [178]. The mutator mouse or the MFN1 knockout mouse individually survive into adulthood, however crosses between the two resulted in embryonic lethality [136]. In this case it is plausible that fusion exerts a protective effect through its ability to “dilute” mutation-containing mtDNA via content mixing and therefore preserve mitochondrial function.

It remains unclear how defects in fusion contribute to the molecular mechanism of deletion formation. Deletions may conceivably accumulate at an increased rate either because of impaired mtDNA replication or because of increased mtDNA damage. It has been speculated that the primary purpose of mitochondrial fusion is to enable the sharing of contents between two mitochondria. Indeed, it has been published that matrix proteins and mtDNA are transferred between fused mitochondria [179]. Conversely, mitochondria from dual MFN knockout cells were found to have increased protein heterogeneity compared to their wild-type counterparts [136]. It may therefore be that the loss of content mixing associated with defective fusion impairs mtDNA replication through an imbalance of replisome components [134]. This could then promote replication stalling and copy-choice recombination, leading to increased deletion formation. Alternatively, it is possible that increased mtDNA damage in the absence of fusion promotes the formation of deletion-containing mtDNA molecules via double-strand break formation. However, a key limitation in the idea of mtDNA repair underlying the formation of deletions is that at this stage the proteins that are responsible for DNA repair have not been identified as being present in mitochondria.

It is also conceivable that deletions accumulate in the absence of fusion because their removal is impaired. However, the observation that defects in fusion are associated with an accumulation of mtDNA mutations and deletions appears counterintuitive, as it would be expected that a fragmented network would be optimal for selective mitophagy to clear dysfunctional mitochondria away. Indeed, in a *Drosophila melanogaster* model of mtDNA heteroplasmy, the knockdown of MFN promoted the mitophagy of fragmented mitochondria and a reduction in the mutant mtDNA load [180]. Similarly, a perpetually fused network due to the inhibition of fission by DRP1 or FIS1 in human cells was associated with a shift in heteroplasmy towards mutant mtDNA, possibly because of reduced mitophagy, although this was not addressed [181]. As such, it appears that the accumulation of mtDNA mutations and deletions in the absence of fusion is not related to a reduction in mitophagy.

Collectively, these studies demonstrate that IMM and OMM fusion have an important role in maintaining the integrity of mtDNA and coordinating its replication. However, the relationship is complex and further study is certainly required. It seems likely that the role of fusion in facilitating content mixing between mitochondria is important to maintain the balance of proteins required for mtDNA replication [134,136]. There is also evidence to suggest that fusion also has a protective role against the effects of ROS and DNA damage [161,177]. This, coupled with an imbalance of replisome proteins leading to replication stalling/slippage, could underlie the onset of mutations and deletions associated with fusion defects, as well as contribute to mtDNA copy number loss.

## 4. The Relationship between mtDNA and Mitochondrial Cristae Structure

**Figure 3:**
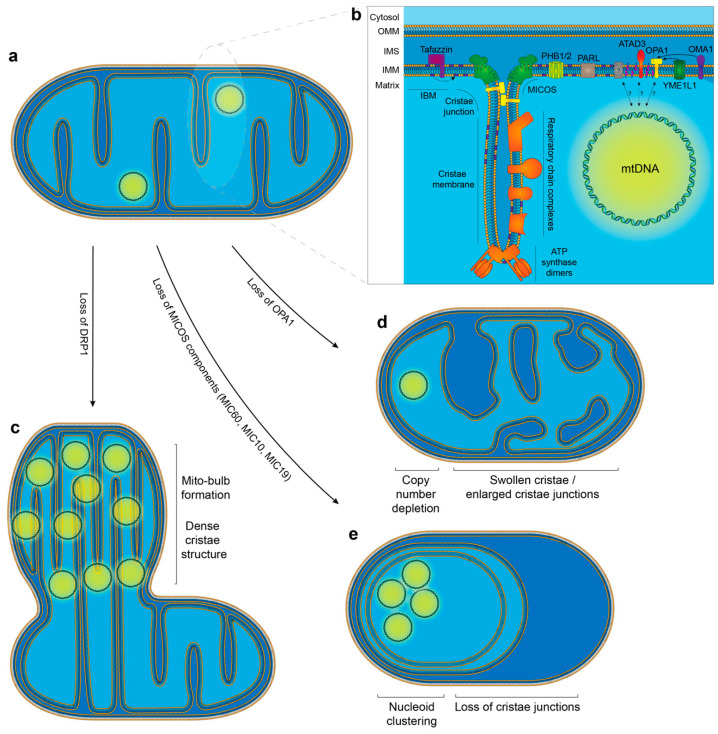
The relationship between cristae modulators and mtDNA organisation. (**a**) Schematic overview of a normal mitochondrion. (**b**) Enlarged cristae region depicting the spatial arrangement of key proteins that are necessary for dictating cristae structure and shape, as well as the organisation of the respiratory chain complexes. Proteins that are potentially responsible for the tethering of mtDNA to the IBM are also highlighted. (**c**) Loss of the fission factor DRP1 has been associated with the formation of mito-bulb structures. These are regions of dense cristae structure which harbour a number of clustered mtDNA molecules. (**d**) The loss of IMM fusion and cristae shaping protein OPA1 results in mitochondria that display enlarged cristae junctions and a perturbed cristae structure. In addition, mtDNA copy number is reduced. (**e**) The loss of MICOS components (MIC60, MIC10 and MIC19) has been associated with a complete loss of cristae junctions and the formation of cristae in concentric circles. Nucleoid clustering is evident following the loss of MICOS components.

The IMM is a complex structure that can be subdivided into two further regions: the inner boundary membrane (IBM) and the cristae (Figure 3b). The IBM runs parallel to the OMM and houses proteins responsible for localising and importing proteins into the matrix, as well as inserting and assembling proteins into the IMM [182,183]. The IBM is divided at regions where the IMM is folded inwards to form cristae structures. Cristae are functionally active structures that contain the respiratory chain complexes and the ATP synthase [184]. At points where the cristae joins the IBM, the cristae narrows to form a cristae junction (approximately 20–40 nm in diameter) which allows the separation of the intracristal space from the intermembrane space between the IMM and OMM [185,186,187]. The mitochondrial contact site and cristae organising system (MICOS) complex is located at these junctions and plays a key role in maintaining their structure.

It has long been appreciated that mtDNA is located close to the IMM and to the cristae structure. It has been recently demonstrated using correlative 3D super-resolution fluorescence and electron microscopy that nucleoids are found within cristae regions of the mitochondria [68]. Sophisticated super-resolution imaging has now revealed that nucleoids are typically located in the voids that form between groups of cristae rather than being embedded within the cristae structure itself [188]. This is in line with the observation that the size of the nucleoid, at approximately 100 nm, is greater than the gaps between cristae, which are found tightly packed together [53,54,188]. It is postulated that these voids where nucleoids are located may function to provide space for transcription, replication and segregation to occur [68,188]. In this section we will summarise current data on cristae structure and modulation, and its relationship with mtDNA maintenance and segregation.

### 4.1. The Importance of Lipids in the IMM and Their Role in mtDNA Tethering

The capacity for mtDNA to freely diffuse around the mitochondrial network is limited, due in part to the relatively large size of the nucleoid and to the high density of proteins located within the matrix and cristae regions [53,69,189]. There is also evidence that has led to the suggestion that mtDNA is tethered to the IMM [67,69,70,190,191]. However, at this stage the factors responsible remain poorly understood.

Several studies have highlighted the importance of the lipid composition of the IMM to mtDNA attachment, replication and organisation. The composition of phospholipids in the IMM is tightly regulated. Phospholipids have a hydrophobic lipid domain tail which is larger than their charged hydrophilic head groups; this configuration forms a rough conical shape which pulls head groups together, inducing membrane tension and facilitating the bending of the IMM to form cristae invaginations [192,193]. Cardiolipin has an important role in stabilising IMM proteins such as the respiratory complexes [194]. Alterations in the level of cardiolipin have been associated with mitochondrial dysfunction, mtDNA loss and the formation of abnormal cristae structures [121,123]. Similarly, in mammalian cells the removal of the IMM phospholipid phosphatidylethanolamine has comparable effects; mitochondrial function is reduced coupled with obvious swelling of mitochondria which lack distinct cristae structures [195].

It has been demonstrated in both yeast and mammalian cells that cardiolipin is physically associated with the nucleoid [120]. Furthermore, as discussed earlier cardiolipin is a key constituent of the IMM and has a role in mitochondrial fission and fusion. As such, the role of cardiolipin in mtDNA maintenance is multi-faceted. The loss of cardiolipin is associated with a reduced capacity to correctly segregate and guide nucleoids to daughter mitochondria. In addition, this defective IMM-nucleoid association coupled with defects in mitochondrial dynamics leads to a lack of mtDNA inheritance between replicating cells resulting in a dysfunctional respiratory phenotype in daughter cells [120,121,122].

The composition of the mitochondrial IMM is subject to a relatively low ratio of cholesterol to phospholipids [196]. Cholesterol has been implicated in nucleoid organisation as its modulation either through pharmacological inhibition or supplementation prompts the formation of aggregated nucleoids in human fibroblasts [171]. The majority of mitochondrial cholesterol is clustered in specialised structures that span the IMM and OMM [197]. TWINKLE-containing nucleoids were shown to be associated with these cholesterol structures in a complex that also contains ATAD3. ATAD3 is an essential protein that is anchored into the IMM, with its C-terminal AAA ATPase domain located in the mitochondrial matrix while its N-terminus interacts with the OMM where it is present at ER-mitochondria contact sites [62,169,197,198,199]. It has been proposed that these cholesterol-rich sites provide a platform for mtDNA replication, and act as an attachment site at ER-mitochondria junctions to allow for the coordinated distribution of mtDNA. As discussed earlier, ATAD3 has been demonstrated to have a regulatory role in mitochondrial morphology and dynamics and has been implicated in nucleoid organisation [167,168,169,170]. ATAD3 also has an important role in maintaining IMM architecture as the disruption of ATAD3 leads to impaired transfer of cholesterol from the ER into the mitochondria and lipid metabolism [171,200]. The knockout of *ATAD3* in skeletal muscle has been associated with alterations in cholesterol metabolism and the progressive formation of mtDNA deletions and copy number depletion [173]. As discussed earlier, the formation of these mtDNA rearrangements has been associated with stalling of the replication machinery and a loss of cristae structure. It has been suggested that ATAD3 may interact with OPA1 or the MICOS complex to stabilise cristae structure [173]. Collectively, it is clear that ATAD3 is an important regulator of mitochondrial dynamics, mtDNA attachment, maintenance and organisation. However, at this stage it is not entirely clear whether these effects are directly associated with the loss of ATAD3 or due to cholesterol-mediated alterations in the IMM structure as a consequence of the role of ATAD3 in the uptake of cholesterol to the mitochondria [200].

In addition to ATAD3, OPA1 has also been implicated as having a potential role in the attachment of nucleoids to the IMM [135]. OPA1 is embedded in the IMM, co-immunoprecipitates with nucleoids and as discussed earlier, its deletion has profound effects on nucleoid organisation [77,135,136]. Thus, it has been suggested that OPA1 may have a role in membrane attachment of the nucleoid (Figure 3b). Loss of either ATAD3 or OPA1 has been shown to cause significant aberrations in cristae structure [161,197,201].

The structure of proteins and lipids within the IMM is also supported by a group of evolutionarily conserved scaffolding proteins termed prohibitins. Prohibitin 1 and 2 are essential proteins that form high molecular weight ring-shaped heterooligomers in the IMM. They have also been found to associate with nucleoids which prompts speculation that they may be involved in tethering of nucleoids to the IMM [57]. It has been demonstrated that the prohibitins can influence mtDNA copy number through their interactions with TFAM [202]. Cultured cells lacking the prohibitins display impaired cardiolipin maturation, loss of cristae structure and disorganisation of nucleoids [203,204,205]. It has also been demonstrated that the effects of prohibitin 2 depletion upon cristae structure are dependent upon OPA1, a known modulator of cristae structure [204].

### 4.2. Modulators of Cristae Structure

Mitochondrial cristae are dynamic structures that can modulate their shape in response to various physiological conditions. As they are the primary site of the OXPHOS machinery, it is important that the folding of the IMM into cristae structures facilitates the most efficient manner of producing ATP. Recent work has found that individual cristae act as autonomous bioenergetic units, highlighting the importance of their structural integrity [206]. Cristae structure is dictated by a variety of “cristae-shaping proteins” (Figure 3b). A key mediator of cristae architecture is the MICOS complex. The MICOS complex consists of at least the subunits MIC10, MIC12, MIC13, MIC19, MIC25, MIC26, MIC27 and MIC60 [207]. Each of these proteins has a specific role in shaping the cristae structure. MIC60 is a core component of the complex and is associated with the formation of cristae junctions and contact sites with the OMM [208,209,210]. MIC10 can also bend membranes and is known to be responsible for forming hairpin structures in the IMM [211,212]. MIC13 has also been recently recognised as being essential for maintaining the stability of the MICOS complex and cristae junction formation [212]. MIC26 has a role in cristae junction formation, and both MIC26 and MIC27 are necessary to maintain normal cristae architecture [213,214].

The F_1_F_0_ ATP synthase is embedded in the IMM and is localised to the cristae tips, where it exists in a dimer confirmation. These dimers are also assembled into oligomers. The presence of these dimers prompts a bending of the surrounding IMM lipid bilayer, highlighting their importance as mediators of cristae structure [215,216]. As such, the loss of the F_1_F_0_ ATP synthase is associated with a loss of cristae invaginations [216]. However, the F_1_F_0_ ATP synthase may also act indirectly to maintain cristae morphology, as it has also been recognized that the F_1_F_0_ ATP synthase interacts with the MICOS complex [217]. It has also been reported that oligomerisation of F_1_F_0_ ATP synthase dimers is promoted by OPA1, which also has a role in cristae shaping [218].

The role of OPA1 in cristae shaping is reflected in its ability to define the diameter and width of cristae junctions [209]. It has been demonstrated in yeast that the OPA1 homologue MGM1 is required to maintain cristae structure by tethering to other OPA1 molecules on the opposing IMM [219]. Indeed, it has been observed that high molecular weight multimers of OPA1 stabilise and induce the formation of tight cristae junctions, whereas lower-order OPA1 oligomers are associated with increased cristae junction and lumen width [209]. The remodeling of cristae is mediated by the integral membrane protease PARL. Processing by PARL produces a short, intramembrane-soluble form of OPA1 that binds with the membrane-bound form to maintain tight cristae junctions [220]. There is some evidence that OPA1 has a broader regulatory role and can adjust mitochondrial respiration by modulating cristae shape. It has been reported that in response to hypoxia there is an increase in the abundance of OPA1 oligomers that prevent cristae remodeling, thereby acting to enhance mitochondrial respiration [221]. It has also been shown that OPA1 can interact with metabolic sensors in response to starvation, acting to adjust the cristae shape to maintain ATP production [222]. High molecular weight OPA1 multimers have also found to be associated with MIC60 and F_1_F_0_ ATP synthase [209,218,223]. Further work will be required to understand the extent to which OPA1 dictates cristae shape alone, and how it functions as part of an interactive network with other cristae shaping proteins.

A number of other proteins have been observed to have an impact on cristae structure, but for which the precise mechanisms are less well understood. MCL1 has been found to have a role in preserving cristae ultrastructure and the maintenance of oligomeric ATP synthase [224]. The loss of proteins that interact with cardiolipin have also been found to disrupt cristae architecture. Specifically, the prohibitins and UQCC3 have all been demonstrated to bind to cardiolipin, and their loss is associated with alterations in cristae structure [204,225,226].

### 4.3. The Relationship between Cristae Structure and mtDNA

A number of studies indicate that there is an interesting but poorly-understood link between mtDNA and cristae ultrastructure. Dissecting this relationship is problematic because of the role that mtDNA-encoded proteins, such as components of the ATP synthase, play in maintaining cristae structure. For example Rho-0 (ρ0) fibroblasts, which are devoid of mtDNA, display sparse cristae structures and swollen mitochondria compared to mtDNA-containing cells [227,228,229]. Embryos lacking TFAM also lack mtDNA and display abnormal cristae structure [230]. Similarly, tissue-specific knockout of TFAM in developed animals has also been shown to ablate cristae structure [231,232]. In cultured cells, the transient depletion of mtDNA using the replication inhibitor 2′-3′-dideoxycytidine also leads to a loss of cristae structure [77]. Other data has suggested a direct role for mtDNA in maintaining cristae structure. The loss of DRP1 is associated with the formation of structures filled with very densely packed cristae and clustered nucleoids termed “mito-bulbs” (Figure 3c) [77]. The formation of these cristae-rich mito-bulbs in DRP1 knockdown cells was not impacted when mitochondrial translation was inhibited using chloramphenicol. However, the depletion of mtDNA prior to DRP1 knockdown prevented the formation of mito-bulbs. This suggests that mtDNA itself may be important for determining cristae architecture rather than its gene products. These data highlight the need for further study to understand how mtDNA contributes to the maintenance of normal cristae structure.

The loss of *OPA1* has been associated with a reduced mtDNA copy number, both in model systems and in patients harbouring *OPA1* mutations [135,161,233]. OPA1-deficient cultured cells also show a distinct loss of cristae structure (Figure 3d) [201,234]. Similarly, in mice, both the mutation of *Opa1* in the heart and deletion of *Opa1* in the skeletal muscle was found to cause the loss of cristae structures [161,235,236]. Other in vivo models in which mtDNA content was reduced, such as the deletion of *Mfn2* in Purkinje cells or the dual knockout of *Mfn1 & 2* in skeletal muscle, have also been shown to be associated with sparse cristae structures. Whilst these observations support the idea that the loss of mtDNA is linked with the disruption of cristae structure, it is likely that the relationship is not this simple. For example, one study noted that the loss of cristae structure in *Opa1* mutant mice occurred prior to the reduction in mtDNA content [235], although this is not surprising because of the key role of OPA1 in maintaining cristae junctions [209].

Analysis of the relationship between mtDNA and cristae structure is also complicated by the fact that a number of interventions which alter mtDNA content also impact on the balance of fission and fusion rates of mitochondria, and therefore the proper organisation of nucleoids around the network. There is also evidence to suggest that normal cristae structure is required to facilitate fission and fusion [210]. The MICOS complex can be destabilized by the loss of the core protein MIC60. This results in a striking phenotype of enlarged mitochondria that display abnormal circular cristae, commonly described as “concentric rings” or an “onion-like structure”, alongside an almost complete loss of cristae junctions (Figure 3e) [210,237]. It has also been reported that the loss of MIC60 causes a reduction in the copy number of mtDNA (139). The downregulation of other MICOS subunits such as MIC19 and MIC10 also leads to a disruption of cristae structure [210]. The silencing of *MIC19* and *MIC60* led to the formation of enlarged and disorganised nucleoids in both mammalian cells and yeast, accompanied by a reduction in mtDNA transcription [210,238]. Similarly, it has been noted that mutations in *CHCHD10*, a constituent of the MICOS complex, leads to a loss of cristae junctions and structure. Furthermore, this was also shown to be linked with the formation of mtDNA deletions, as well as a reduction in nucleoid number without an overall effect on copy number [239].

It is worth noting that it is often difficult to determine whether apparently enlarged nucleoids represent one single enlarged nucleoid consisting of multiple physically joined (catenated) mtDNA molecules, or a group of nucleoids clustered in close proximity. An interesting aspect of the enlargement or clustering of nucleoids associated with the loss of *MIC60* is that it was not reported to lead to the formation of cristae-enriched mito-bulb structures as are seen following *DRP1* knockdown [77]. However, it is likely that these cells are unable to form dense cristae regions as a consequence of the fundamental role of MIC60 and the MICOS complex in shaping cristae [207]. Interestingly, *MIC60* knockdown cells showed a normal balance of fission and fusion compared to wild-type cells, but this occurred at a reduced rate with DRP1 being one of the mitochondrial dynamics proteins observed to be downregulated. When DRP1 was overexpressed to promote mitochondrial fragmentation, the presence of enlarged nucleoids associated with the loss of MIC60 was partially reverted [210]. Furthermore, work in yeast has found that when *MIC60 (FCJ1)* is knocked out in conjunction with *DRP1 (DNM1)* the nucleoids are further enlarged compared to the loss of *MIC60* alone [238].

Cristae junctions have also been implicated in enabling nucleoid distribution. Unlike *MIC60* deletion, the knockout of F_1_F_0_ ATP synthase dimerization factors does not promote nucleoid aggregation or affect the number of cristae junctions. However, its knockout does reduce the number of cristae tips. The dual knockout of *MIC60 (FCJ1)* and dimerization partners of F_1_F_0_ ATP synthase results in an increased number of ring-like cristae structures that rescues the nucleoid aggregation phenotype associated with *MIC60* deletion alone [238]. This finding indicates that cristae structure is likely important for partitioning nucleoids to prevent their aggregation.

Addressed collectively, these studies support the idea that normal cristae structure is required to support the even distribution of mtDNA nucleoids around the mitochondrial network (Figure 3). Firstly, dynamic cristae structure is required to facilitate normal rates of fission and fusion, which as discussed earlier are key mediators of nucleoid dispersal. Secondly, cristae can also prevent the aggregation of nucleoids by forming partitioning structures. This is in logical agreement with the observation that nucleoids are generally located in voids between groups of cristae structures [188]. At this stage, it is unclear whether nucleoid partitioning is a precursor to the distribution of nucleoids around the network via fission. Therefore, further study will be necessary to understand the intricate interplay between these two events.

### 4.4. Cristae Remodelling by Independent Fission and Fusion of Cristae Membranes

It has been speculated for some time that cristae are dynamic structures that can undergo remodelling of their membranes in response to various physiological conditions. However, studying these remodelling events in real-time has been particularly challenging until recently. A number of groups have developed methods that allow for the visualisation of cristae structures in live cells using super-resolution microscopy [188,206,240]. Recent work has established that cristae within the same mitochondrion can have different membrane potentials, thus demonstrating that cristae can behave as independent bioenergetic units [206]. This work suggests that within a mitochondrion cristae can functionally isolate themselves from one another, and it is postulated that this mechanism can prevent individual dysfunctional cristae from disrupting membrane potential in the broader mitochondrial network. Further work has revealed that cristae membranes within a mitochondrion undergo fission and fusion events in a MICOS-dependent manner [241]. Specifically, this study provides evidence for a model in which MIC60 is evenly distributed along the IBM, acting as a docking or scaffolding platform to facilitate the formation of cristae junctions following the recruitment of MIC10. The recruitment of other subunits to complete the MICOS complex allows cristae junctions to fully form. Both cristae junctions and cristae membranes are dynamic, and it was observed that cristae junctions have the capacity to split and merge. Cristae membranes can detach from one cristae junction, and fuse with either the same cristae junction or another cristae junction, such as one on the opposing IBM. It was demonstrated that the merging of cristae membranes is associated with changes in membrane potential at that region, and that these events can facilitate content mixing between distinct cristae compartments [241].

The fact that cristae continuously undergo remodelling and have the capacity to function independently raises the question of how these processes are involved in maintaining the integrity of mtDNA, as well as its segregation and normal distribution. This novel ability to study cristae dynamics in live cells allows an unprecedented opportunity for the field to address some of these experimentally difficult questions. For example, what is the importance of dynamic cristae membranes in maintaining mtDNA integrity by content mixing? Other directions may include understanding the interplay between overall mitochondrial dynamics and cristae dynamics, as well as addressing how the modelling of cristae membranes impacts the segregation and movement of mtDNA around the mitochondrial network.

### 4.5. Genetic Defects Associated with Perturbed Mitochondrial Cristae Structure and Diseases

Mammalian MICOS comprises seven protein subunits of which defects in two encoding genes have been linked to human disease so far. Mutations in *MICOS13* (aka *QIL1*) cause severe infantile mitochondrial disease characterised by failure to thrive, microcephaly, truncal hypotonia, spasticity and cerebellar atrophy, lactic acidosis and 3-methylglutoconic aciduria (3-MGA) [242,243,244]. Subclinical hepatic involvement manifesting with persistently abnormal liver function tests was also reported. Altered mitochondrial cristae morphology was evident in liver tissue and fibroblasts, but the mitochondrial ultrastructure in muscle appeared normal. Multiple mitochondrial respiratory chain deficiencies were identified in skeletal muscle and liver biopsies, and isolated complex IV deficiency was present in fibroblasts. However, there was no evidence of mtDNA deletions or depletion in muscles and fibroblasts [242,243,244]. More recently, a mutation in *APOO*, encoding MIC26, has been reported to cause an X-linked disease associated with developmental delay, hypotonia, autistic spectrum disorder, gastrointestinal symptoms, lactic acidosis and abnormal carnitine profile [245]. The pathogenicity of the *APOO* variant was supported by work with fibroblasts and a fly model.

Homozygous mutations in the PINK1 gene, encoding for PTEN-induced kinase 1, were first identified in three consanguineous families with early-onset Parkinsonism through linkage analysis in 2004 [246]. Overall, PINK1 mutations account for less than 10% of autosomal recessive Parkinson’s disease (PD) [247]. Both Parkin and PINK1 have been shown to play crucial roles in mediating the mitophagy process [248]. More recently, PINK1 was found to maintain cristae junctions by the phosphorylation of the MICOS subunit MIC60 in both *Drosophila melanogaster* and human neurons [249]. Furthermore, mutations within the mitochondrial targeting sequence of MIC60 were evident in a subset of PD patients. The introduction of these patient mutations into a *Drosophila melanogaster* model disrupted the ability for MIC60 to localise to mitochondria and prompted the formation of abnormal cristae junctions. Furthermore, the overexpression of MIC60 in a *PINK1^−/−^* model of PD compensated for the loss of PINK1-mediated phosphorylation and rescued both cristae defects and mitochondrial function [249]. In addition, mutations in the mitochondrial targeting sequence of CHCHD2 have been identified as a risk factor for PD and Lewy body disease [250].

CHCHD10 is a protein located in the mitochondrial inter-membrane space and it has been demonstrated to play a role in the maintenance of cristae integrity [239]. Heterozygous mutations in *CHCHD10* were first linked to motor neuron disease and frontotemporal dementia, myopathy and hyperkalemia, impaired respiratory chain function and multiple mtDNA deletions in the skeletal muscle in a large family of French origin, and in a Spanish family (none of the family members underwent muscle or skin biopsy) in 2014 [251]. Whilst mutations of CHCHD10 display a mitochondrial disease that resembles motor neuron disease, a large consortium of motor neuron disease (amyotrophic lateral sclerosis) patients (*n* = 4365) and healthy controls (*n* = 1832) from seven countries subsequently demonstrated that pathogenic *CHCHD10* variants are exceptionally rare [252]. Therefore, in pure forms of amyotrophic lateral sclerosis it is not necessarily associated with *CHCHD10* mutations.

Prominent extra-neurological disease has been observed following the disruption of several proteins implicated in the maintenance of cristae structure, for example, hypertrophic cardiomyopathy occurs in patients harbouring mutated forms of *ATAD3A* and *TAZ* (Barth syndrome) [253,254], and 3-MGA in *ATAD3A*, *TAZ*, *MICOS13* and *ATP5F1E* [243,253,255,256].

## 5. Conclusions

The structure and dynamics of the mitochondrial membranes are required to maintain both the integrity of mtDNA and well as its distribution within the mitochondrial network. Mitochondrial membranes make a poorly understood contribution to mtDNA replication, the impairment of which manifests either as an inability to maintain a sufficient number of copies of mtDNA, or as rearrangements of the genome. Following replication, the dynamics of mitochondria are also required for the segregation of mtDNA, and the disruption of mitochondrial dynamics can lead to the clustering of nucleoids within cells. The direct association between mtDNA and the respiratory chain means that an uneven distribution of nucleoids can lead to a mosaic pattern of respiratory activity within cells, which may represent an under-appreciated molecular contributor to mitochondrial pathologies. Observations from the clinic highlight the importance of these mechanisms, with mutations in genes associated with fusion, fission and cristae structure manifesting mainly in severe neurological disorders frequently associated with mtDNA depletion or rearrangements.

Work from the laboratory puts forward the notion that mitochondrial fusion is necessary to maintain adequate levels of mtDNA replication, likely highlighting the importance of content mixing for maintaining a proper stoichiometry of replisome components between mitochondria. Indeed, it is plausible that this protein heterogeneity may prompt replication stalling resulting in the formation of deletions and rearrangements of mtDNA. The reduction in mitochondrial DNA copy number observed in conjunction with the disruption of fusion therefore likely occurs due to reduced rates of replication.

Defects in mitochondrial fission on the other hand have less of an association with the onset of mtDNA rearrangements or a reduction in mtDNA copy number in the laboratory. However, mutations of fission genes still result in severe neurological disorders in the clinic. As mitochondrial function is often observed to be normal when fission is impaired, the mechanism underlying these clinical phenotypes is not entirely clear at this stage. It is understood that mitochondrial fission is necessary for mitochondrial quality control which can facilitate the accumulation of dysfunctional mitochondria. Furthermore, fission is essential for the even allocation of nucleoids around the mitochondrial network and to subsequent daughter cells. Disruption of fission leads to the clustering of nucleoids. Indeed, the presence of mtDNA is intricately linked to the formation of normal cristae architecture. The clustering of nucleoids is associated with the formation of dense cristae regions in the form of mito-bulb structures, and the loss of mtDNA is often correlated with the loss of cristae structure. The data available suggests that dynamic cristae are necessary to facilitate normal rates of mitochondrial fission and fusion, as well as allow for the partitioning of mtDNA. As such the interactions between dynamics, cristae and mtDNA organisation are tightly interwoven and interdependent. It is therefore likely that if each of these processes is not tightly regulated then there are subsequent downstream effects that disrupt the delicate balance and prompt the onset of mitochondrial dysfunction.

## Abbreviations

3-MGA	3-methylglutoconic aciduria
AD	autosomal dominant
AR	autosomal recessive
ATAD3	ATPase family AAA domain-containing protein 3
ATP	adenosine triphosphate
CK	creatine kinase
CMT	Charcot-Marie-Tooth
COX1	cytochrome c oxidase 1 subunit
CPEO	chronic progressive external ophthalmoplegia
CSF	cerebrospinal fluid
DOA	dominant optic atrophy
DD	developmental delay
DRP1	dynamin-related protein 1
ER	endoplasmic reticulum
ESRF	end-stage renal failure
FIS1	mitochondrial fission 1 protein
FSGS	focal segmental glomerulosclerosis
GTP	guanine triphosphate
HSP	heavy strand promoter
IBM	inner boundary membrane
IMM	inner mitochondrial membrane
IMS	intermembrane space
LA	lactic acidosis
LS	Leigh syndrome
LSP	light strand promoter
MCL1	induced myeloid leukemia cell differentiation protein
MEF	mouse embryonic fibroblast
MERRF	myoclonic epilepsy and ragged-red fibres
MFN	Mitofusin
MICOS	mitochondrial contact site and cristae organising system
MID49	mitochondrial dynamics protein of 49kDa
MitoPLD	mitochondria-localised phospholipase D
MRC	mitochondrial respiratory chain
mtDNA	mitochondrial DNA
MTERF1	mitochondrial transcription termination factor 1
mtSSB	mitochondrial single stranded DNA-binding protein
NCR	noncoding region
OA	optic atrophy
OMA1	overlapping with the M-AAA protease 1 homolog
OMM	outer mitochondrial membrane
OPA1	optic atrophy 1
OriH	origin of H-strand replication
OriL	origin of L-strand replication
OXPHOS	oxidative phosphorylation
PA	phosphatidic acid
PARL	presenilin-associated rhomboid-like
PD	Parkinson’s disease
POLγ	DNA polymerase-γ
POLRMT	mitochondrial RNA polymerase
rRNA	ribosomal RNA
RRFs	ragged-red fibres
RTA	renal tubular acidosis
SCA28	spinocerebellar ataxia type 28
SNHL	sensorineural hearing loss
TEFM	mitochondrial transcription elongation factor
TFAM	mitochondrial transcription factor A
TFB2M	mitochondrial transcription factor B2
tRNA	transfer RNA
VLCFA	very long chain fatty acid
WM	white matter
YME1L1	YME1 like 1 ATPase

## Appendix A

**Table A1:** Summary of clinical phenotype, extra-neurological involvement and integrity of mitochondrial DNA associated with the genetic defects of mitochondrial dynamics.

	Gene	Protein	Protein Function	Inheritance	Clinical Phenotype	Extra-Neurological	mtDNA Integrity	References
**Regulators of Mitochondrial Dynamics**	DRP1	Dynamin-related protein 1	Mitochondrial fission	AD	Microcephaly, abnormal brain development, OA, LA, elevated VLCFA; DD, refractory epilepsy; isolated DOA (OPA5)	None	EM & confocal microscopy study showing concentric cristae structure in patient-derived fibroblasts [97]	[92,97,257,258]
							CIV deficiency in muscle [257]	
							Elongated mitochondria in optic nerve and RGC layer without axonal degeneration in Dnm1l^+/−^ mice [258]	
							No reports of mtDNA deletions or depletion	
	DNM2	Dynamin 2	Mitochondrial fission	AD	Centronuclear myopathy; dominant intermediate CMT neuropathy type B associated with neutropenia and cataract; CPEO, facial weakness, neck flexor weakness; severe cardiomyopathy and centronuclear myopathy	Cardiac, neutropenia	COX negative fibres and multiple mtDNA deletions in skeletal muscle [103]	[100,101,102,103,259,260]
	GDAP1	Ganglioside Induced Differentiation Associated Protein 1	Mitochondrial fission	AR	CMT4A (early onset with rapid progression demyelinating neuropathy)	None	No reports of mtDNA deletions or depletion	[93,106,108,109]
				AD	CMT2RV (axonal neuropathy with hoarse voice and diaphragmatic weakness)			
	INF2	Inverted Formin, FH2 And WH2 Domain Containing	Mitochondrial fission	AD	CMT, dominant, intermediate type, E (CMTDIE) with SNHL and renal phenotype (FSGS, proteinuria and ESRF)	Renal	No reports of mtDNA deletions or depletion	[94,104,105,261]
	MFF	Mitochondrial Fission Factor	Mitochondrial fission	AR	DD, pyramidal signs and neuropathy; Leigh-like disease with infantile spasm, OA and neuropathy	None	Normal mitochondrial respiratory chain activities in muscle; significant branching of mitochondria in patient-derived fibroblasts [98]	[95,98,107]
	SLC25A46	Solute Carrier Family 25 Member 46	Mitochondrial fission	AD	DOA and CMT2; hereditary motor and sensory neuropathy, Type VIB (HMSN6B)	None	Abnormal mitochondrial cristae following knockdown in fibroblasts [99]	[96,99,262,263]
							Mitochondrial elongation following knockdown in Zebrafish, mtDNA integrity not assessed [262]	
				AR	Progressive myoclonic ataxia, OA and neuropathy; LS; pontocerebellar hypoplasia and apnoea			
							No reports of mtDNA deletions or depletion	
	OPA1	OPA1 Mitochondrial Dynamin Like GTPase	IM fusion/cristae shaping	AD	DOA (most common genetic cause); DOA plus syndrome (CPEO, ataxia, axonal neuropathy, deafness); syndromic Parkinsonism and dementia	Cardiac	Abnormal cristae in OPA1^+/−^ mouse model [264]	[141,143,174,265,266,267,268,269,270]
				AR	Ataxia, early-onset OA, gastrointestinal dysmotility; Behr syndrome (OA and ataxia); Infantile encephalomyopathy, HCM and OA; Leigh-like imaging changes		COX negative fibres and multiple mtDNA deletions in cases of heterozygous mutation [141,174]	
							Multiple respiratory chain deficiencies and mtDNA depletion in muscle with recessive inheritance [265]	
	MFN2	Mitofusin 2	OM fusion	AD	CMT2A; DOA plus; DD, progressive weakness, failure to thrive, axonal sensorimotor neuropathy, chorea and elevated CSF lactate	Lipoma	Multiple mtDNA deletions and mtDNA depletion detected in muscles and fibroblasts [154]	[154,157,176,271,272,273,274,275]
				AR	Severe, early-onset axonal neuropathy, OA; multiple symmetric lipomatosis, LA and axonal neuropathy		RRFs, COX deficient fibres, altered mitochondrial morphology and multiple mtDNA deletion detected in muscle [176]	
							COX deficient fibres, ultrastructural changes and mtDNA depletion	
	FBLX4	F-Box And Leucine Rich Repeat Protein 4	OM fusion	AR	Encephalopathy, microcephaly and persistent LA; encephalomyopathy, abnormal WM changes, LA, dysmorphism, RTA, seizures	Renal (RTA)	Multiple respiratory chain deficiencies and mtDNA depletion in patient-derived fibroblasts [155]	[155,276,277,278,279]
							Normal MRC enzymes but mtDNA depletion in muscle [276]	
							mtDNA depletion, reduced respiratory function and ultrastructure abnormalities in muscle, reduced respiratory function in fibroblasts [277]	
	MSTO1	Misato Mitochondrial Distribution And Morphology Regulator 1	Interacts with mitochondrial fusion machinery	AR	Myopathy, elevated CK (1200–4500 IU/L), pigmentary retinopathy, scoliosis and cerebellar ataxia and atrophy	None	Normal MRC enzymes, reduced mtDNA copy number in muscle; mitochondrial fragmentation in patient-derived fibroblasts [280]	[280,281,282]
				AD	Myopathy, cerebellar ataxia and non-specific neuropsychiatric symptoms			
	YME1L1 (YME1)	YME1 like 1 ATPase	OPA1 processing	AR	Intellectual disability, DD, OA, hearing impairment, ataxia, LA, leukoencephalopathic changes on MRI,	None	Altered cristae ultrastructure in muscle, mitochondrial fragmentation in patient-derived fibroblasts [283]	[283]
	SPG7	SPG7 Matrix AAA Peptidase Subunit, Paraplegin	OPA1 processing	AR	Spastic paraplegia 7; spastic ataxia; cerebellar ataxia; CPEO and myopathy;	None	Altered mitochondrial ultrastructure; multiple respiratory chain deficiencies in muscle [147,284]	[147,148,153,284,285]
							RRFs, COX-deficient fibres and multiple mtDNA deletions; normal mtDNA copy number in muscle; reduced network size in patient-derived fibroblasts [153]	
	AFG3L2	AFG3 Like Matrix AAA Peptidase Subunit 2	OPA1 processing	AD	Spinocerebellar ataxia 28 (SCA28); CPEO	None	RRFs, COX deficient fibres and multiple mtDNA deletions in muscle [152]	[152,286,287,288]
**Regulators of Cristae Structure**	MICOS13 (MIC13; QIL1)	Mitochondrial Contact Site And Cristae Organizing System Subunit 13	Stabilises MIC60 and MIC10 in the MICOS Complex	AR	Failure to thrive, acquired microcephaly, truncal hypotonia, limb spasticity cerebellar atrophy, LA, 3-MGA, elevated liver transaminases and myoclonic seizures	Liver	Normal histology and MRC activities in muscle; low MRC enzymatic activities and abnormal cristae in liver [243]	[243,244]
							Multiple respiratory chain deficiencies in muscle and liver [244]	
	APOO (MIC26)	Apolipoprotein O	Regulatory role in MICOS complex	X-linked	DD, myopathy, LA, cognitive impairment, autistic features, abnormal acylcarnitidine profile	None	Abnormal ultrastructure in fibroblasts and a Drosophila Melanogaster model [245]	[245]
	CHCHD10	Coiled-Coil-Helix-Coiled-Coil-Helix Domain Containing 10	Stabilises MICOS complex	AD	Frontotemporal dementia and amyotrophic lateral sclerosis; late onset spinal motor neuronopathy and elevated CK; myopathy, elevated CK and lactate; CMT2	None	RRFs, COX deficient fibres, multiple respiratory chain deficiencies, abnormal assembly of CV, multiple mtDNA deletions in muscle, reduced fusion and loss of cristae structure in patient-derived fibroblast [251]	[239,251,252,289,290,291,292]
							Mildly increased COX deficient fibres and occasional RRFs [290] and multiple mtDNA deletions [290]	
							Multiple respiratory chain deficiencies (worst with CIV), RRFs, lipid accumulation and abnormal circular cristae ultrastructure [291]	
							Loss of mitochondrial cristae junctions with impaired mitochondrial genome maintenance and inhibition of apoptosis in patient-derived fibroblasts [239]	
	PINK1	PTEN Induced Kinase 1	Mitophagy/Interacts with MIC60 to regulate CJs	AR	Parkinson disease 6 (PARK6)	None	Complex I deficiency in Drosophila Melanogaster model [293]	[246,293,294,295,296,297]
	ATP5F1E F_1_F_o_-ATP synthase (sub unit E)	ATP Synthase F1 Subunit Epsilon	Cristae shaping/ATP Production	AR	LA, 3-MGA, mild mental retardation and development of peripheral neuropathy	None	Complex V deficiency in patient-derived fibroblasts [256]	[256]
	ATP5F1A F_1_F_o_-ATP synthase (sub unit a)	ATP Synthase F1 Subunit Alpha	Cristae shaping/ATP production	AR	Intrauterine growth retardation, microcephaly, hypotonia, pulmonary hypertension, failure to thrive, encephalopathy, and heart failure; neonatal-onset encephalopathy with intractable seizures, extensive signal changes involving WM, basal ganglia and thalamus	Cardiac	Multiple respiratory chain deficiency and mtDNA depletion in muscle [298]	[298,299]
							Complex V deficiency in muscle [299]	
**IMM Structure**	ATAD3A	ATPase Family AAA Domain Containing 3A	mtDNA and cristae organisation	De novo & AR	Global DD, hypotonia, OA, axonal neuropathy, mildly elevated lactate, seizures, 3-MGA and hypertrophic cardiomyopathy; fatal congenital pontocerebellar hypoplasia with a simplified gyral pattern; cerebellar atrophy with dystonia and ataxia	Cardiac	Abnormal cristae in Drosophila Melanogaster and patient-derived fibroblasts [253]	[171,253]
							mtDNA abnormalities in patient-derived fibroblasts [171]	
	TAZ	Tafazzin	Organisation of cardiolipin in the IM	X-linked	Severe infantile cardiomyopathy (left ventricular noncompaction, Barth syndrome with dilated cardiomyopathy), sudden cardiac death, myopathy, neutropenia, growth failure, 3-MGA, facial dysmorphism	Cardiac, neutropenia	Altered mitochondrial ultrastructure, stacked and circular cristae, in patient-derived lymphoblasts [300]	[254,255,300,301,302,303,304]
